# Polyphenol oxidases exhibit promiscuous proteolytic activity

**DOI:** 10.1038/s42004-020-0305-2

**Published:** 2020-05-15

**Authors:** A. Biundo, V. Braunschmid, M. Pretzler, I. Kampatsikas, B. Darnhofer, R. Birner-Gruenberger, A. Rompel, D. Ribitsch, G. M. Guebitz

**Affiliations:** 1 Institute of Environmental Biotechnology, University of Natural Resources and Life Sciences (BOKU), Konrad Lorenz Straße 22, 3430 Tulln, Austria; 2 Department of Biosciences, Biotechnology and Biopharmaceutics, University of Bari, Via Edoardo Orabona, 70125 Bari, Italy; 3 Austrian Centre for Industrial Biotechnology (ACIB), Konrad Lorenz Straße 22, 3430 Tulln, Austria and Petersgasse 14, 8010 Graz, Austria; 4 Universität Wien, Fakultät für Chemie, Institut für Biophysikalische Chemie, Althanstraße 14, 1090 Wien, Austria; 5 Medical University of Graz, Diagnostic and Research Institute of Pathology, Neue Stiftingtalstraße 6, 8010 Graz, Austria; 6 BioTechMed-Graz, Mozartgasse 12/II, 8010 Graz, Austria; 7 Vienna University of Technology, Institute for Chemical Technologies and Analytics, Getreidemarkt 9/164, 1060 Vienna, Austria

**Keywords:** Oxidoreductases, Enzyme mechanisms, Biocatalysis

## Abstract

Tyrosinases catalyse both the cresolase and catecholase reactions for the formation of reactive compounds which are very important for industrial applications. In this study, we describe a proteolytic activity of tyrosinases. Two different tyrosinases originating from mushroom and apple are able to cleave the carboxylesterase EstA. The cleavage reaction correlates with the integrity of the active site of tyrosinase and is independent of other possible influencing factors, which could be present in the reaction. Therefore, the cleavage of EstA represents a novel functionality of tyrosinases. EstA was previously reported to degrade synthetic polyesters, albeit slowly. However, the EstA truncated by tyrosinase shows higher degradation activity on the non-biodegradable polyester polyethylene terephthalate (PET), which is a well-established environmental threat.

## Abstract (web summary)

Tyrosinases are an industrially significant class of polyphenol oxidase. Here, two tyrosinases are shown to cleave a specific peptide bond in a carboxylesterase, yielding a truncated product with higher catalytic activity than the full-length enzyme.

## Introduction

In nature, a limited number of enzymes catalyse hundred-thousands of reactions in a cell. One approach of nature to deal with the disproportion between enzymes and biochemical reactions, is the expression of “multifunctional enzymes”^1^. These enzymes are proteins that perform several distinct enzymatic reactions. Multifunctionality is very important for industrial applications and our understanding of enzymes. In this study, we describe a proteolytic functionality of polyphenol oxidases (PPOs).

Tyrosinases (EC 1.14.18.1 and EC 1.10.3.1), catechol oxidases (EC 1.10.3.1) and aurone synthases (EC 1.21.3.6) constitute the PPO family, which belongs to the type-III-copper protein class^2–4^. PPOs contain a dinuclear copper centre, which consists of two Cu ions (CuA and CuB) coordinated by the τ nitrogen atoms of three conserved histidine residues for each Cu ion^5,6^. PPOs are ubiquitous in the different domains of life^7^. They are important in biosynthetic pathways, such as the formation of brown colour in fruits after the loss of cell compartmentalization and the contact with atmospheric oxygen^8^.

**Fig. 1:**
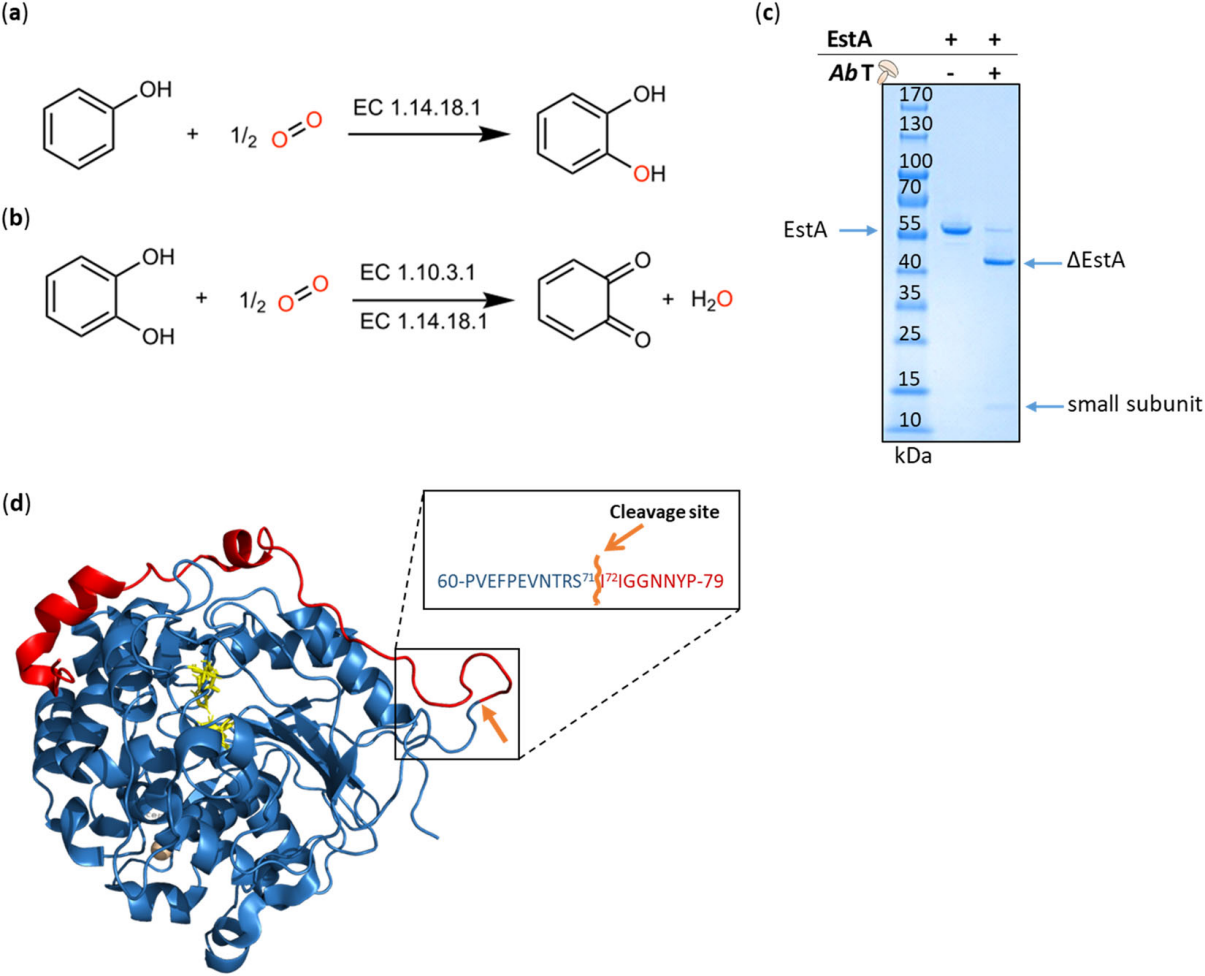
Activities of tyrosinases. Tyrosinases catalyse reactions from monophenols to o-quinones through monophenolase (**a**) and diphenolase (**b**) activity. Incubation of the commercial tyrosinase from *A. bisporus* (*Ab*T) with the carboxylesterase EstA led to a cleavage of the latter into a big (ΔEstA) and a small subunit (**c**). EstA was cleaved at a specific site between Ser71 and Ile72. The 71 amino acids cleaved off form an α-helix and a loop on the surface of EstA (PDB 5AH1) (released N-terminal part in red; ΔEstA in blue, cleavage site in orange, catalytic triad of the active site in yellow sticks) (**d**).

Most eukaryotic PPOs are expressed as latent pro-enzymes containing an active domain and a shielding C-terminal domain. Plant PPOs contain also an N-terminal signal peptide, which is thought to direct the enzyme into the lumen of thylakoids, whereas the C-terminal domain of PPOs covers the active site. The activation of the enzymes can be achieved via the cleavage of the C-terminal domain by proteases, at acidic pH or in the presence of detergents. In nature, the most probable activation mechanism is proteolytic maturation, although this is still under investigation^9,10^. In vitro activation of latent PPOs (lPPOs) is carried out by site-specific cleavage with proteases yielding active forms of PPOs (aPPOs) or chemically by sodium dodecyl sulphate (SDS), which partially unfolds the C-terminal domain, making the active site accessible. Members of the tyrosinase family possess cresolase and catecholase activity (Fig. 1a). The cresolase activity (monophenolase activity, EC 1.14.18.1), is responsible for the *ortho*-hydroxylation of monophenols and subsequent oxidation to *o*-quinones. On the other hand, the catecholase activity (diphenolase activity, EC 1.10.3.1), is specific for the oxidation of *o*-diphenols to *o*-quinones^11,12^. The highly reactive *o*-quinones can undergo further non-enzymatic rearrangements leading to complex polymers such as melanin^13^. The mechanistic division of PPOs into tyrosinases (cresolase and catecholase activity) and catechol oxidases (only catecholase activity) is still under consideration^14^. Moreover, aurone synthase can catalyse the formation of aurones from chalcones through hydroxylation and oxidative cyclization using a mechanism similar to tyrosinases^15,16^.

Tyrosinases have been investigated for many different biotechnological applications^17^. They are widely used for cross-linking of proteins, because of their ability to convert surface-exposed tyrosines of polypeptides into *o*-quinones, which in turn react spontaneously through 1,4-additions with the side chain of amino acids that contain a nucleophilic group^18^. This cross-linking activity has made tyrosinases very important in the food industry in order to modify the structure and texture of food proteins. In a similar attempt to immobilize a carboxylesterase from *Clostridium botulinum* (EstA, formerly published as Cbotu_EstA) to a suitable carrier, we have recently discovered an unusual activity of tyrosinases. Incubation of EstA with certain tyrosinases resulted in a complete cleavage of the EstA into a larger and a smaller subunit. The carboxylesterase EstA has been identified in the past to hydrolyse synthetic polyesters such as polybutylene adipate-*co*-terephthalate (PBAT)^19^. Processing of the carboxylesterase by tyrosinase resulted in a truncated version of EstA (ΔEstA) lacking 71 amino acids at the N-terminus. Interestingly, ΔEstA showed drastically improved activities on soluble and insoluble substrates, such as the recalcitrant polyester polyethylene terephthalate (PET), which strengthens their environmental and industrial relevance.

In this study, we test several PPOs in their active form towards EstA. Cleavage activity is present in three out of the five tested PPO-containing products. The cleavage is specific for a defined site between Ser71 and Ile72, which is not known as a typical protease cleavage site. To our knowledge this intermolecular cleavage activity was never reported before for members of the PPO family.

## Results

### Polyphenol oxidases exhibit proteolytic activity

In this study, the proteolytic activity was first discovered using a commercial tyrosinase preparation from *Agaricus bisporus* (*Ab*T) (Fig. 1c), initially intended for the purpose of enzyme immobilization on carriers. Although tyrosinase from *A. bisporus* is well studied and commercially available, enzyme formulations may consist of a mixture of several isoenzymes and may contain stabilizers and additional, undeclared enzymatic activities^20^. Hence, in order to confirm proteolytic activity of pure isoenzymes, the recombinantly produced fourth isoform of *A. bisporus* tyrosinase (*Ab*PPO4), was investigated in detail^21–24^. On L-tyrosine, the activated form of the recombinant tyrosinase (a*Ab*PPO4) displayed an activity of 2.8 U mg^−1^. Upon cleavage with a*Ab*PPO4, analysis of the ΔEstA by liquid chromatography coupled to mass spectroscopy (LC-MS/MS) unveiled a specific non-tryptic cleavage site between Ser71 and Ile72, embedded in the motive NH_2_-VNTR**SI**IGGN-COOH of EstA (Supplementary Fig. 1 and Supplementary Tables 1 and 2). Interestingly, in silico studies of the cleavage site showed that the motif is not specific for any known protease^25^ except for thermolysin. Thermolysin, however, was predicted to cut at 103 further sites throughout the sequence of EstA^26^, which was not observed by SDS-PAGE for the cleavage by PPOs (Supplementary Table 3).

The 3D structure of ΔEstA was modelled for investigation of the cleaved part (Fig. 1d). The model was based on the crystal structure of EstA recently solved at 1.2 Å (PDB 5AH1)^19^. The structure revealed that the 71 amino acids cleaved off by the tyrosinase form a small *α*-helix and a long, solvent exposed loop that span over the active site of the enzyme.

The truncated enzyme exhibited a 1.9-fold increased activity, from 268 ± 5 U mg^−1^ to 523 ± 17 U mg^−1^, on the model substrate *para-*nitrophenyl butyrate (*p*NPB) compared to the uncleaved enzyme (Supplementary Fig. 2). Previous studies showed that the N-terminal *α*-helix might hinder the entrance of the substrate into the active site of EstA. This *α*-helix is positioned between the *α*-helices of the lid-domain, which are needed for interfacial activation (Fig. 1d)^27^.

**Fig. 2:**
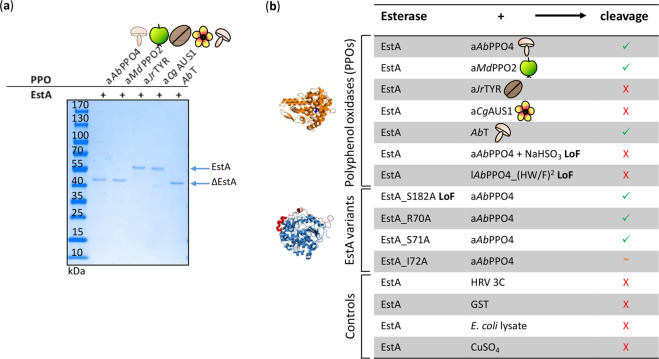
Cleavage with different PPOs. PPOs from *A. bisporus* (commercial: *Ab*T, recombinant: a*Ab*PPO4), *M. domestica* (a*Md*PPO2) *J. regia* (a*Jr*TYR) and *C. grandiflora* (a*Cg*AUS1) were tested for cleavage activity on EstA (**a**). Various experiments were carried out to characterize the reaction, summarized in **b**.

In order to identify a proteolytic activity conserved throughout the PPO family, experiments with other PPOs were conducted (Fig. 2a). Three members of the tyrosinase family were investigated, i.e., the recombinant a*Ab*PPO4 (from common mushroom)^21^, the recombinant PPO2 from *Malus domestica* (a*Md*PPO2 from “Golden Delicious” apple)^28^ and the tyrosinase isolated from *Juglans regia* leaves (a*Jr*TYR from walnut)^29,30^. In addition, another member of the PPO family, the aurone synthase purified from *Coreopsis grandiflora* petals (a*Cg*AUS1 from large-flowered tickseed)^16,31^, was included in the study. The aurone synthase a*Cg*AUS1 is classified as a catechol oxidase as it is not reactive on classical tyrosinase substrates tyrosine or tyramine, however, it can hydroxylate its natural substrate isoliquiritigenin^16^. Recombinant a*Md*PPO2 exhibited the same proteolytic activity on EstA as a*Ab*PPO4 (Fig. 2a, lane 2 and 3, respectively). The two other members of the PPO family, a*Jr*TYR and a*Cg*AUS1, did not exhibit any cleavage of EstA (Fig. 2a, lane 4 and 5, respectively). The distinctive behaviour of the different PPOs is difficult to account for, due to high similarity of the structures of all tested enzymes and especially of their active sites. Hence, the recombinantly produced tyrosinase a*Ab*PPO4^21^ was used for further characterization of the cleavage (Fig. 2b).

### Cleavage was independent of possible impurities

Although EstA was consistently stable during expression^19^, the effect of other substances involved in the downstream processing of a*Ab*PPO4 that might cleave EstA, were investigated. Both enzymes, a*Ab*PPO4 and EstA, were expressed in *Escherichia coli* BL21(DE3). First, the influence of *E. coli* BL21(DE3) cell-lysate on the purified EstA was examined to exclude any possible interference with protein stability. During 30 h of incubation, the enzyme EstA was stable in the presence of *E. coli* BL21(DE3) cell-lysate (Supplementary Fig. 3a).

Recombinant tyrosinases were purified via the N-terminal affinity tag glutathione-S-transferase from *Schistosoma japonicum* (GST tag), which was cleaved off by the cysteine protease 3C from human rhinovirus serotype 14 (HRV 3C) after purification. GST and HRV 3C are active on the tripeptide glutathione and on a specific recognition sequence (LEVLFQ/GP), respectively^32,33^. GST and HRV 3C could potentially contribute to the cleavage of EstA. To exclude this possibility, the two enzymes, GST and HRV 3C, were separately incubated with EstA at the assay conditions. Neither of these compounds showed an effect on EstA (Supplementary Fig. 3a). For the expression of the recombinant tyrosinases, copper sulphate was supplemented to the medium during expression, to permit the correct insertion into the tyrosinase active site. This salt was likewise tested and did not show any cleavage of EstA (Supplementary Fig. 3a).

### Esterase activity does not contribute to cleavage

EstA showed the potential to hydrolyse synthetic polyesters^19^. Peptide (amide) bonds differ from ester bonds in the nitrogen atom bound to the carbonyl carbon. The presence of the nitrogen atom in amides confers higher resistance to cleavage since the so-called nitrogen inversion mechanism must take place for the bond to be hydrolysed^34^. However, the serine hydrolase enzyme family can cleave peptides^35^ and EstA as a member might have this activity as well^19^. The stability of EstA was tested in each experiment, carrying the reactions out with EstA alone.

To further exclude any auto-proteolysis activity from the natural active site of the enzyme, an inactive EstA variant was constructed (EstA_S182A) and tested. Variant EstA_S182A contains a substitution of the nucleophilic Ser182 of the catalytic triad to alanine that inactivates the enzyme. Inactivity was tested by esterase activity assays on *p*NPB (Supplementary Fig. 4). This variant was incubated with a*Ab*PPO4 and showed identical cleavage pattern to the wild-type EstA (Supplementary Fig. 3b). Therefore, a contribution of EstA to its proteolytic cleavage could be excluded.

### Inhibition of tyrosinase activity inhibits the cleavage

In order to characterise the proteolytic activity of PPOs and to potentially identify the presence of a second active site, inhibition of their natural activity, i.e., polyphenol oxidase activity, was examined. Sulphites are well-known irreversible inhibitors of PPO activity. The inactivation of PPOs is achieved by binding of the sulphite ion to a histidine residue that coordinates one of the copper ions of the active site, which inhibits the activity irreversibly^36^. In this study, the loss of PPO activity of a*Ab*PPO4 was verified by an activity assay with dopamine (Supplementary Fig. 5). Indeed, inactivation of a*Ab*PPO4 through sodium bisulphite effectively inhibited the proteolysis of EstA (Supplementary Fig. 3c) confirming the involvement of the PPO’s active site in proteolytic cleavage.

Furthermore, to confirm these results, the gene encoding for the latent *Ab*PPO4 (l*Ab*PPO4) was mutated, to generate loss-of-function variants. The loss-of-function l*Ab*PPO4 variants, l*Ab*PPO4_(HF)^2^ and l*Ab*PPO4_(HW)^2^, were constructed to disrupt the coordination of the copper ions in the active site. Accordingly, histidines at positions 91 and 251 were exchanged for phenylalanine in l*Ab*PPO4_(HF)^2^ and histidines at positions 57 and 282 to tryptophan in l*Ab*PPO4_(HW)^2^. None of the inactive *Ab*PPO4 variants cleaved EstA (Supplementary Fig. 3d). Hence, these results confirmed the multifunctionality of a*Ab*PPO4.

### Substitutions in the cleavage site affect the cleavage

**Fig. 3:**
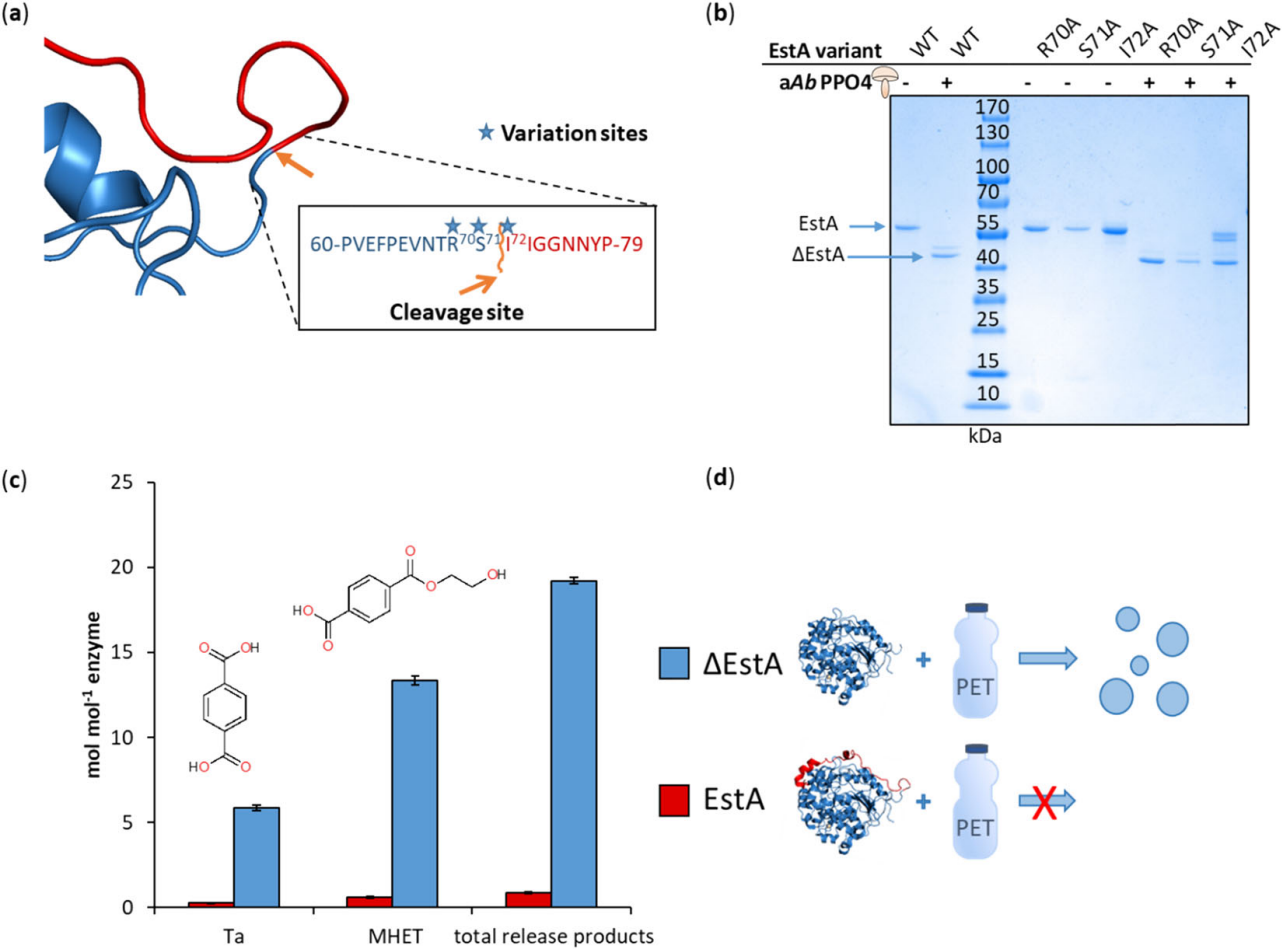
Characterization of EstA variants. EstA variants with substitutions in the cleavage site (R70A, S71A and I72A) were created (**a**) to elucidate the contribution of these residues. The cleavage of these variants with a*Ab*PPO4 was monitored and compared to the cleavage of wild type (WT) EstA (**b**). HPLC analyses of the hydrolysis products showed a higher increase in MHET and Ta after hydrolysis with the truncated (ΔEstA) compared to the full-length EstA. Measurements were performed in triplicates and the standard deviation is given in the error bars (**c**). EstA hydrolysed PET more efficiently when cleaved by tyrosinases (**d**).

Proteases are highly specific for certain amino acid sequences. The physicochemical properties of the amino acids surrounding the cleavage site can influence the specificity and activity of the protease. The peptide motif of the identified cleavage site in EstA was Arg70-Ser71-Ile72 at positions P2-P1-P1’ (Fig. 3a), according to the Schechter and Berger nomenclature^37^. These three amino acids are diverse in their physicochemical properties. Arginine is positively charged with a long side chain, whereas serine is relatively small and polar, but uncharged. Isoleucine, moreover, is uncharged, but bulkier and non-polar. In order to characterize the influence of these amino acids on the proteolysis by PPOs, we exchanged these residues in single substitutions for alanine. Alanine is a small, uncharged and non-polar residue and differs substantially from all of the exchanged residues. The variants created were specifically: EstA_R70A, EstA_S71A and EstA_I72A. Cleavage of these variants with a*Ab*PPO4 conveyed diverse results (Fig. 3b). The exchange of Arg70 by alanine led to the biggest change in physicochemical properties, decreasing size and charge. This variant was completely cleaved by a*Ab*PPO4, as shown for wild type EstA (Fig. 3b, lane 7). Proteolysis of EstA_S71A resulted in a similar pattern (Fig. 3b, lane 8). Interestingly, cleavage of the variant EstA_I72A took place, but it was not complete after 30 h (Fig. 3b, lane 9). Proteolysis of this variant yielded less cleavage product in comparison to that of the wild-type enzyme, after 30 h. For this variant, a band at a molecular weight slightly lower than EstA was observed. This band could be an intermediate between EstA and the truncated EstA. Analyses of this product could help elucidate the underlying mechanism. From these results, we can deduce that the amino acid in position P1’ is important for the cleavage reaction and the comparatively long side chain of Ile72 is involved in the mechanism.

### Cleavage increases hydrolytic activity of the esterase

EstA cleaved by PPOs was truncated by 71 amino acids. The truncation opened the entrance to the active site and exposed a hydrophobic patch on the surface of the enzyme^27^. Hydrophobic regions on the surface can increase the sorption to hydrophobic substrates, such as polyesters^38^. Previously, we have reported the increased activity of the recombinantly expressed truncated version of EstA on the soluble substrate *p*NPB and on the insoluble polyester PET^27^. This truncated EstA missed the same 71 amino acids as did the ΔEstA cleaved by PPOs. The carboxylesterase EstA, truncated by proteolytic cleavage with tyrosinases, exhibited drastically higher activities than both the un-cleaved and the recombinant truncated enzyme. Analysis of PET hydrolysis showed that the total amount of release products, terephthalic acid (Ta) and mono-2-hydroxyethyl terephthalate (MHET), was 32-fold higher than the products released by the full-length EstA (Fig. 3c, d). On the other hand, the EstA recombinantly produced as the truncated carboxylesterase released only 8-fold more hydrolysis products than the wild-type^27^. This could be due to differences in folding caused by the high hydrophobicity present on the surface during the expression of the truncated version. The shielding of hydrophobic patches during expression is important for the correct folding of proteins. Protein folding is driven by the minimalization of energy and the maximization of Van-der-Waals forces. Therefore, hydrophobic residues are usually located on the inside of proteins or in cavities^39,40^.

## Discussion

In this work, five PPOs from plant and fungal kingdoms of life were tested for their proteolytic activity on the carboxylesterase EstA. Out of the five PPOs, three enzyme products (commercial *Ab*T, a*Ab*PPO4 and a*Md*PPO2) were found to proteolytically cleave EstA. *Ab*PPO4 and *Ab*T are tyrosinases from common mushroom (*A. bisporus*), while *Md*PPO2 is a tyrosinase from apple (*M. domestica*) and therefore they are from different kingdoms of life. Other active PPOs from plants, a*Jr*TYR from walnut (*J. regia*) and a*Cg*AUS from large-flowered tick-seed (*C. grandiflora*) did not show any cleavage of EstA at the tested conditions, despite their high similarity to a*Ab*PPO4 and a*Md*PPO2. A comparison of 3D-models did not reveal crucial differences (Supplementary Fig. 6). Thorough analyses of the cleavage mechanism were performed with different forms of *Ab*PPO4.

*Ab*PPO4 cleaved EstA at a specific site between Ser71 and Ile72. The cleavage at this site resulted in the truncation of EstA, removing 71 amino acids at the N-terminus. As a result, the truncated EstA showed a two-fold increased activity towards the soluble substrate *p*NPB and a 32-fold increased hydrolysis of the polymer PET, compared to the full-length enzyme. Although the activity of ΔEstA was drastically improved, its activity is still lower than the activity of PETase from *Ideonella sakaiensis*^41^. EstA is, however, an anaerobic enzyme, expressed under aerobic conditions. Anaerobic conditions might increase its activity on PET. Therefore the comparison of the two enzymes is difficult. Analysis of auto-proteolysis and activity of impurities, potentially present in the enzyme preparations, showed that the cleavage of EstA was dependent on the presence of tyrosinases.

In our study, we described for the first time the cleavage of a full-length protein by tyrosinases. Therefore, it is probable that this cleavage followed a different mechanism compared to previously reported studies^42^. Recently, it was reported that an isoform of the *Md*PPO2 (namely *Md*PPO1) from *M. domestica* showed self-cleavage due to a peptide present in the linker between the C-terminal domain and the active site. In the mentioned study, the self-cleavage was deactivated only upon deletion of the entire peptide, while mutagenesis experiments did not suppress the self-cleavage of *Md*PPO1^9^. The (auto)proteolytic process of PPOs does not resemble the mechanism of any of the known proteases like serine proteases, cysteine proteases or metalloproteases. PPO-induced proteolysis is quite slow (hours to days), while proteases usually cleave their substrate in seconds or minutes. Proteases often contain catalytic triads (serine^43^ or cysteine proteases^44^) or metals like zinc (metalloproteases^45^) at their active sites, which function as activators for water, thereby allowing for hydrolytic peptide bond cleavage. In PPOs, however, similar catalytic motifs are not present and therefore their proteolytic activity cannot be explained with the known protease mechanisms.

Models of *Ab*PPO4 show a single active site. Furthermore, the presented results that focused on the inactivity of *Ab*PPO4, showed that the cleavage was correlated with tyrosinase activity. Therefore, we can conclude that the active site of *Ab*PPO4 plays a crucial role in the presented reaction. The described results point to a catalytic promiscuity of *Ab*PPO4 for proteolytic activity.

Substitutions of the residues Arg70, Ser71 and Ile72 of EstA to alanine suggested the importance of the amino acid Ile72, which is directly adjacent to the cleavage site. Further substitutions would be needed to determine the exact influence of residues, which are proximal and distal to the cleavage site. Furthermore, substitutions to amino acids, other than alanine, could identify side chains that are important for the reaction.

This is the first time an intermolecular proteolysis of folded proteins by tyrosinases has been reported, to our knowledge. Multifunctionality of enzymes is very attractive for industrial applications. Further investigations of this newly found activity of PPOs might not only lead to new applications, but could help understand the promiscuity of this very important and useful class of enzymes.

## Methods

### Chemicals and reagents

Pre-stained protein marker IV was obtained from Peqlab (Germany) and the protein determination kit was from Biorad (USA). All other chemicals and reagents were purchased from Sigma Aldrich (Germany) or Carl Roth (Germany) and were at least of analytical grade. The commercially available *Ab*T, sodium bisulphite as well as the substrates *p*NPB and dopamine hydrochloride were procured from Sigma Aldrich. Q5 High-Fidelity DNA Polymerase, *Sap*I and T4 DNA ligase were purchased from New England Biolabs (NEB, Germany) and *Esp*3I from Fisher Scientific (Austria). The pGEX-6P-1 (GE Healthcare, Austria) and pENTRY-IBA51 (IBA, Germany) vectors were used as templates and nucleotide primers were synthesised by Sigma-Aldrich.

### Construction and cultivation of bacterial strains

The strain *E. coli* BL21(DE3) was used for the expression of all esterases in lysogeny broth (LB) medium supplemented with the appropriate antibiotic. The esterase EstA and its variants were cloned into the expression vector pET26b(+) and expressed in LB medium containing 40 µg mL^−1^ kanamycin sulphate. Media, bacterial strains and plasmids for the production of *Ab*PPO4 and *Md*PPO2 were as previously described^21,28^.

For the generation of enzymatically inactive variants of *Ab*PPO4 the gene encoding the latent form of the tyrosinase^21^ was cloned into the pENTRY vector. Therefore the required *Sap*I recognition sequences were added to both ends using the two primers pENTRY-*lAbPPO4*_fwd and pENTRY-*lAbPPO4*_rev. The amplicon was cut-ligated^46^ into pENTRY-IBA51 using *Sap*I and T4 DNA ligase. Mutagenesis was carried out in two steps introducing one point mutation each, applying the Q5® Site-Directed Mutagenesis Kit (NEB, Germany). The four pairs of mutagenic primers (Supplementary Table 4) targeted one histidine at the active centre each (H91F and H251F for l*Ab*PPO4_(HF)^2^, H57W and H282W for l*Ab*PPO4_(HW)^2^). Sequences were verified by Sanger sequencing (Microsynth, Austria). The genes with the newly introduced bulky amino acids at the copper-coordination site were subcloned into the expression vector pGEX-6P-SG (*vide infra*) by cut-ligation with the type IIS restriction endonuclease *Esp*3I and T4 DNA ligase.

The adaptation of the pGEX-6P-1 vector to the StarGate® cloning system (IBA, Germany) was carried out by Q5® Site-Directed Mutagenesis in two steps. In the first step the two recognition sequences for *Esp*3I in pGEX-6P-1 were removed. One of them was removed by introducing a silent point mutation in the recognition sequence located inside the *lac*I gene (primers pGEX-6P-1_C4295A_fwd & rev) and the other by deletion of a 121 bp segment containing the multiple cloning site (primers pGEX-6P-1 -> pGEX-6P-SG_fwd & rev). The primers for the deletion step were also used to introduce 28 bp containing the two *Esp*3I recognition (6 bp) and cutting (5 bp) sequences required for the StarGate® cloning system as well as a single *Sma*I recognition site (6 bp) located between the two nonpalindromic *Esp*3I recognition sequences.

### Expression and purification of enzymes

EstA wild-type and variants were expressed and purified as described by Perz et al.^19^. Genes encoding the EstA variants were codon optimized for the *E. coli* codon usage and synthesized in a pET26b(+) vector by GenScript (USA). The activated forms of a*Ab*PPO4 and a*Md*PPO2 as well as the enzymatically inactive variants l*Ab*PPO4_(HF)^2^ and l*Ab*PPO4_(HW)^2^ were prepared as previously described^21,28^*. Jr*TYR was isolated from walnut leaves and purified applying cation exchange chromatography^29^. *Cg*AUS1 was isolated from the petals of *C. grandiflora* and purified using a series of anion and cation exchange steps^16^.

### Determination of proteolytic activity of polyphenol oxidases

For the proteolysis of EstA and its variants, 4 mM of each esterase variant were mixed with 0.6 µM of PPOs in 0.1 M Tris-HCl buffer pH 6. The final reaction volume was 300 µL. The reactions were carried out at 23 °C and shaking at 300 rpm for 30 h, if not stated otherwise. Each reaction was done at least twice to replicate the results. To characterise the cleavage reactions, various substances were added to the reaction mixture in place of the PPOs. Specifically, 0.5 mM copper sulphate, 0.6 µM GST, 1.3 µM HRV 3 C and 30 µL of the *E. coli* BL21(DE3) cell-lysate were individually added. The *E. coli* lysate was obtained through the lysis of *E. coli* BL21(DE3) by ultrasonication three times for 45 s with an amplitude of 60% and 2 min breaks in between sonication steps. The *E. coli* lysate was prepared by centrifugation at 20,817*g* for 5 min.

Latent *Ab*PPO4 variants, l*Ab*PPO4_(HF)^2^ and l*Ab*PPO4_(HW)^2^, were activated by the addition of 2 mM sodium dodecylsulphate (SDS) to the reaction mixture. To inhibit tyrosinase activity, a*Ab*PPO4 was incubated with 10 mM sodium bisulphite for 1 h at 23 °C. The tyrosinase was subsequently separated from the sodium bisulphite by ultrafiltration with Vivacon 2 ultrafiltration devices with 30 kDa cut-off and a Hydrosart^®^ membrane (Sartorius, Germany) and washing with 400 µL 0.1 M Tris-HCl buffer pH 6. The washing and centrifugation was repeated three times. Inhibition was verified by measurement of the activity on 15 mM dopamine. The activity was measured photometrically at 475 nm (*ε*_475_ = 3.1) in 0.1 M Tris-HCl buffer pH 6 (Supplementary Fig. 7).

*Protein analyses:* Protein concentration was determined by Bradford Assay^47^ with a Bio-Rad Protein determination kit (Bio-Rad, USA) using bovine serum albumin as a protein standard. Photometric measurements were performed with a Tecan plate reader Infinite M200 PRO (Tecan, Switzerland). Proteolytic cleavage of EstA and its variants as well as the purity of freshly produced enzymes was determined with SDS-polyacrylamide gel electrophoresis (SDS-PAGE) according to Laemmli^48^. Precast gels were purchased from Bio-Rad (USA), with 4% stacking and 15% separating gel and were run at 150 V. As molecular mass marker pre-stained protein marker IV (Peqlab, Germany) was used. Gels were stained with 1.25 g L^−1^ Coomassie Brilliant Blue solution containing 30% ethanol and 10% acetic acid. Destaining was performed with the same solution excluding Coomassie Brilliant Blue G 250.

*Biochemical characterization:* The activity of the carboxylesterases was determined spectrophotometrically with the model substrate *p*NPB (100 µM final concentration) in 0.1 M potassium phosphate buffer pH 7 (*ε*_405_ = 8.31 M^−1^ cm^−1^)^49,50^. Enzymatic activity of polyphenol oxidase preparation was tested in 50 mM sodium citrate buffer pH 6.8 at 25 °C with 1 mM l-tyrosine as the substrate and 2 mM SDS for activation of latent enzymes^21^. The resulting formation of dopachrome (*ε*_475_ = 3600 M^−1^ cm^−1^) was monitored photometrically and the enzymatic activity was determined from the linear part of the absorption-time curve. One unit (U) of enzymatic activity was defined as the amount of enzyme catalysing the formation of 1 µmol reaction product per minute under the conditions specified above.

*Mass spectroscopy:* For LC-MS/MS analysis protein bands were excised from gels, reduced, alkylated and tryptically cleaved according to the manufacturer´s instructions (Promega, USA). Peptide extracts were dissolved in 0.3% formic acid, 5% acetonitrile and separated by nano-HPLC (Dionex Ultimate 3000) equipped with a µ-precolumn (C18, 5 µm, 100 Å, 5 × 0.3 mm) and a Acclaim PepMap RSLC nanocolumn (C18, 2 µm, 100 Å, 150 × 0.075 mm) (all Thermo Fisher Scientific, Austria). Samples of 20 µL were injected and concentrated on the enrichment column for 2 min using 0.1% formic acid as isocratic solvent at a flow rate of 5 μL min^−1^. The column was then switched into the nanoflow circuit, and the sample was loaded on the nanocolumn at a flow rate of 250 nl min^−1^ at 60 °C and separated using the following gradient: solvent A: water, 0.1% formic acid; solvent B: acetonitrile, 0.1% formic acid; 0–2 min: 4% B; 2-90 min: 4–25% B; 90–95 min: 25–95% B, 96–110 min: 95% B; 110–110.1 min: 4% B; 110.1–125 min: 4% B. The sample was ionized in the nanospray source equipped with stainless steel emitters (Thermo Fisher Scientific, Austria) and analyzed in a Thermo Orbitrap Velos Pro mass spectrometer in positive ion mode by alternating full scan MS (m/z 380 to 2000) and MS/MS by CID of the 20 most intense peaks. The LC-MS/MS data were analyzed by searching a homemade database containing the protein sequence and common contaminations with Mascot 2.3 (MatrixScience, UK) and Proteome Discoverer 1.3. Carbamidomethylation on cysteine was entered as fixed and oxidation on methionine as variable modification. Detailed search criteria were used as follows: semi trypsin; max. missed cleavage sites: 2; MS/MS ion search with decoy database search included; precursor mass tolerance ±0.05 Da; product mass tolerance ±0.7 Da; acceptance parameters: *p* < 0.05; minimum 2 peptides; ion score cut off: 20 and FDR 1%.

### Degradation of polyester

Amorphous PET films (0.5 × 1 cm) were thoroughly washed^27^. Washed PET films were incubated with 5 µM EstA in 0.1 M potassium phosphate buffer pH 7 at 50 °C and 100 rpm for 120 h. Supernatants were collected and analysed by high performance liquid chromatography (HPLC) (Agilent Technologies, USA)^27^.

### Modelling

Structural data for the enzymes was derived from the PDB using the following entries: 5AH1 (EstA), 5M6B (a*Ab*PPO4), 5CE9 (a*Jr*TYR1), 4Z0Y (a*Cg*AUS1). For a*Md*PPO2 a homology model was prepared using the SWISS-MODEL Server^51^ with the coordinates of *Md*PPO1 (59.6% sequence identity, PDB entry 6ELS) as the template.

## Supplementary information

Supplementary Information

## Data Availability

The datasets generated and/or analysed during the current study are included in the publication and supplymentary information or available from the corresponding author on reasonable request or publicly available on Research Collaboratory for Structural Bioinformatics Protein Data Bank (RCSB PDB; a member of wwPDB; https://www.rcsb.org/) (PDB IDs for individual structures are given in the manuscript).
