# Supplementary material for: Polyphenol oxidases exhibit promiscuous proteolytic activity
Source: Commun Chem. 2020 May 15;3:62. doi: 10.1038/s42004-020-0305-2 (PMC9814219; doi:10.1038/s42004-020-0305-2)
Supplement: Supplementary file 1 — Supplementary Information [file 42004_2020_305_MOESM1_ESM.pdf]

Supplementary Information

Polyphenol oxidases exhibit promiscuous proteolytic activity

A. Biundo[a,b,+], V. Braunschmid[a,c,+], M. Pretzler[d], I. Kampatsikas[d], B. Darnhofer[c,e,f], R. Birner-Gruenberger[e,f,g], A. Rompel[d], D. Ribitsch[a,c,*], G. M. Guebitz[a,c]

[a] *Institute of Environmental Biotechnology, University of Natural Resources and Life Sciences (BOKU), Konrad Lorenz Straße 22, 3430 Tulln, Austria*

[b] *Department of Biosciences, Biotechnology and Biopharmaceutics, University of Bari, Via Edoardo Orabona, 70125 Bari, Italy*

[c] *Austrian Centre for Industrial Biotechnology (ACIB), Konrad Lorenz Straße 22, 3430 Tulln, Austria and Petersgasse 14, 8010 Graz, Austria*

[d] *Universität Wien, Fakultät für Chemie, Institut für Biophysikalische Chemie, Althanstraße 14, 1090, Wien, Austria; http://www.bpc.univie.ac.at*

[e] *Medical University of Graz, Diagnostic and Research Institute of Pathology, Neue Stiftingtalstraße 6, 8010 Graz, Austria*

[f] *BioTechMed-Graz, Mozartgasse 12/II, 8010 Graz, Austria*

[g] *Vienna University of Technology, Institute for Chemical Technologies and Analytics, Getreidemarkt 9/164, 1060 Vienna , Austria*

[+] *These authors contributed equally: Antonino Biundo, Verena Braunschmid*

** Corresponding author: Doris Ribitsch, e-mail: doris.ribitsch@boku.ac.at*

Mass spectrum data

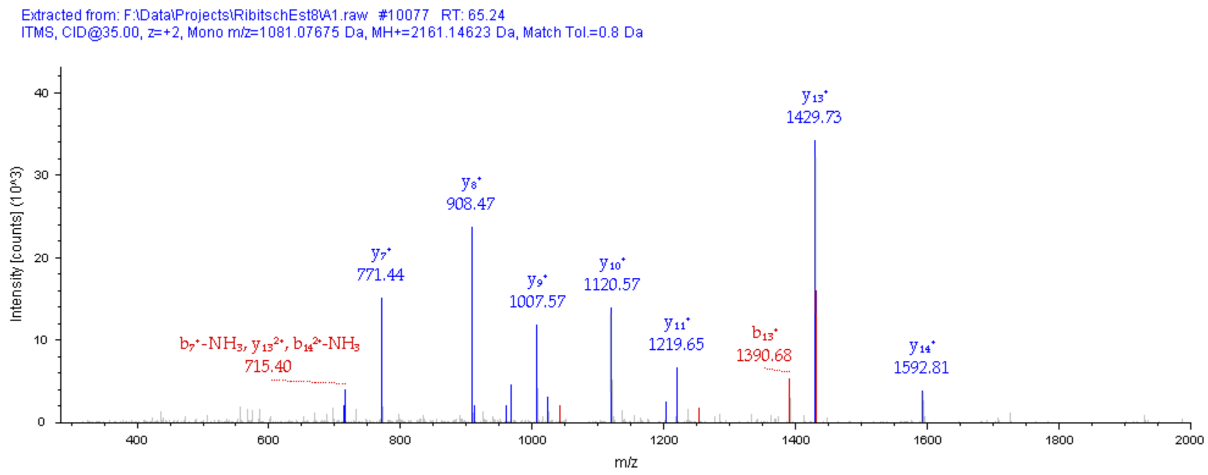

Supplementary Figure 1: **MS/MS spectrum** of the non-tryptic peptide IIGGNNYPIVLVHGFMGFGR (precursor mass error 3.98 ppm, ion score 96, exp. value 5,1399 E-09).

Supplementary Table 1: **Ion table of the MS/MS spectrum** of the non-tryptic peptide IIGGNNYPIVLVHGFMGFGR shown in *Supplementary Figure 1*

#1	b^+	b^{2+}	Seq.	y^+	y^{2+}	#2
1	114.09135	57.54931	I			20
2	227.17542	114.09135	I	2048.05356	1024.53042	19
3	284.19689	142.60208	G	1934.96949	967.98838	18
4	341.21836	171.11282	G	1877.94802	939.47765	17
5	455.26129	228.13428	N	1820.92655	910.96691	16
6	569.30422	285.15575	N	1706.88362	853.94545	15
7	732.36754	366.68741	Y	1592.84069	796.92398	14
8	829.42031	415.21379	P	1429.77737	715.39232	13
9	942.50438	471.75583	I	1332.72460	666.86594	12
10	1041.57280	521.29004	V	1219.64053	610.32390	11
11	1154.65687	577.83207	L	1120.57211	560.78969	10
12	1253.72529	627.36628	V	1007.48804	504.24766	9
13	1390.78420	695.89574	H	908.41962	454.71345	8
14	1447.80567	724.40647	G	771.36071	386.18399	7
15	1594.87409	797.94068	F	714.33924	357.67326	6
16	1725.91459	863.46093	M	567.27082	284.13905	5
17	1782.93606	891.97167	G	436.23032	218.61880	4
18	1930.00448	965.50588	F	379.20885	190.10806	3
19	1987.02595	994.01661	G	232.14043	116.57385	2
20			R	175.11896	88.06312	1

Supplementary Table 2: **Spectral counts of the non-tryptic peptides** starting with IIGGNNY of EstA incubated with *Ab*T and a*Ab*PPO4 as compared to EstA alone (negative control).

	EstA + *Ab*T	EstA (neg. control)	EstA + a*Ab*PPO4
IIGGNNYPIVLVHGFmGFGR	15		13
IIGGNNYPIVLVHGFMGFGR	13		1
IIGGNNYPIVLVHGFMGFGRDELLGYK			
IIGGNNYPIVLVHGFmGFGRDELLGYK			

Prediction of proteases cleaving at cleavage site

Supplementary Table 3: **Proteases predicted to cut in EstA sequence** and number and position of cleavage sites. Predicted by PeptideCutter by Expasy.[1]

Name of enzyme	*No. of cleavages*	*Positions of cleavage sites*
Arg-C proteinase	12	70 91 131 158 161 189 285 290 365 368 420 452
Asp-N endopeptidase	28	22 34 36 40 50 91 104 129 144 231 237 243 267 271 285 292 301 306 324 371 376 383 392 400 424 427 437 441
Asp-N endopeptidase + N-terminal Glu	58	2 12 19 22 24 25 33 34 36 40 43 44 48 49 50 62 65 91 92 104 107 115 129 133 144 147 172 196 199 200 201 207 214 231 237 243 267 271 281 285 288 292 301 306 310 314 324 337 371 376 383 392 400 403 424 427 437 441
BNPS-Skatole	10	100 129 220 274 297 000 000 000 000 000
CNBr	10	1 55 87 183 240 352 363 418 427 441
Chymotrypsin-high specificity (C-term to [FYW], not before P)	38	86 89 97 99 100 118 129 136 138 146 154 159 167 214 220 248 250 263 267 274 287 297 306 319 327 328 329 331 356 366 380 406 415 433 439 440 443 446
Chymotrypsin-low specificity (C-term to [FYWML], not before P)	83	1 33 39 58 82 84 86 87 89 94 95 97 99 100 106 110 115 118 129 135 136 138 146 150 154 156 159 167 177 178 181 183 191 194 195 213 214 220 222 236 239 245 248 250 253 262 263 267 269 271 274 276 287 292 297 306 308 316 319 327 328 329 331 341 344 352 356 362 363 366 380 397 406 415 427 433 439 440 441 443 446 447 450
Clostripain	12	70 91 131 158 161 189 285 290 365 368 420 452
Formic acid	28	23 35 37 41 51 92 105 130 145 232 238 244 268 272 286 293 302 307 325 372 377 384 393 401 425 428 438 442
Glutamyl endopeptidase	30	3 13 20 25 26 34 44 45 49 50 63 66 93 108 116 134 148 173 197 200 201 202 208 215 282 289 311 315 338 404
Hydroxylamine	2	280 407
Iodosobenzoic acid	10	100 129 220 274 297 000 000 000 000 000
LysC	32	5 11 18 19 22 29 47 54 59 98 109 152 153 155 168 175 218 243 259 261 270 277 278 301 321 337 371 378 379 396 411 437

LysN	32	4 10 17 18 21 28 46 53 58 97 108 151 152 154 167 174 217 242 258 260 269 276 277 300 320 336 370 377 378 395 410 436
NTCB (2-nitro-5-thiocyanobenzoic acid)	3	132 204 388
Pepsin (pH1.3)	60	33 38 39 60 64 81 82 85 88 89 94 95 105 106 109 110 134 135 153 178 190 193 194 195 212 214 235 236 238 244 249 250 252 253 262 268 269 270 271 275 276 291 307 308 315 316 328 329 340 341 361 362 397 432 433 445 446 447 449 450
Pepsin (pH>2)	105	33 38 39 60 64 78 81 82 85 88 89 94 95 96 97 98 99 105 106 109 110 118 128 129 134 135 136 137 138 145 146 153 159 166 167 178 190 193 194 195 212 214 219 235 236 238 244 247 248 249 250 252 253 262 266 267 268 269 270 271 273 274 275 276 286 291 296 297 305 306 307 308 315 316 318 319 326 327 328 329 330 331 340 341 357 361 362 366 379 397 405 406 414 415 432 433 438 440 442 443 445 446 447 449 450
Proteinase K	216	2 3 6 9 12 13 16 17 20 21 24 25 26 27 28 30 32 33 34 36 39 40 43 44 45 46 49 50 52 58 60 62 63 64 66 67 69 72 73 78 80 81 82 83 86 89 93 94 95 97 99 100 103 104 106 108 110 112 116 117 118 119 120 121 122 125 129 132 134 135 136 137 138 139 140 143 144 146 148 149 151 154 159 162 163 166 167 170 173 176 178 179 187 188 190 191 192 194 195 197 200 201 202 203 208 210 213 214 215 220 221 224 226 227 229 234 235 236 239 242 245 246 248 249 250 252 253 255 256 257 262 263 266 267 269 271 274 276 282 287 288 289 291 292 296 297 300 303 304 305 306 308 310 311 313 315 316 318 319 320 322 326 327 328 329 331 332 333 335 336 338 340 341 342 346 347 349 355 356 357 359 360 362 366 373 375 376 380 381 386 387 390 397 402 403 404 406 410 412 415 417 421 422 424 429 430 432 433 436 439 440 443 444 446 447 450
Staphylococcal peptidase I	25	3 13 20 25 34 44 49 63 66 93 108 116 134 148 173 197 200 208 215 282 289 311 315 338 404
Thermolysin	104	1 5 11 27 32 38 39 42 57 61 71 72 79 80 81 82 85 86 88 94 102 103 109 111 119 121 124 131 136 138 139 143 150 153 165 169 175 177 178 182 187 190 193 194 209 212 213 220 223 226 235 241 245 249 251 252 255 261 262 265 270 275 287 290 291 295 303 312 319 321 328 334 339 340 345 346 348 351 354 355 359 361 362 374 375 385 386 389 396 402 409 411 416 420 421 426 429 432 435 440 443 445 446 449
Trypsin	44	5 11 18 19 22 29 47 54 59 70 91 98 109 131 152 153 155 158 161 168 175 189 218 243 259 261 270 277 278 285 290 301 321 337 365 368 371 378 379 396 411 420 437 452

Activity of truncated EstA

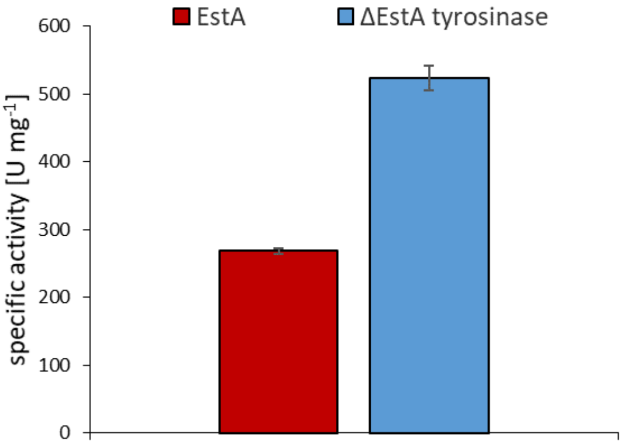

Supplementary Figure 2: **Specific activity of EstA** on 15.75 mM *p*NPB before and after proteolysis by a*Ab*PPO4. Measurements were performed in triplicates and the standard deviations are given as error bars.

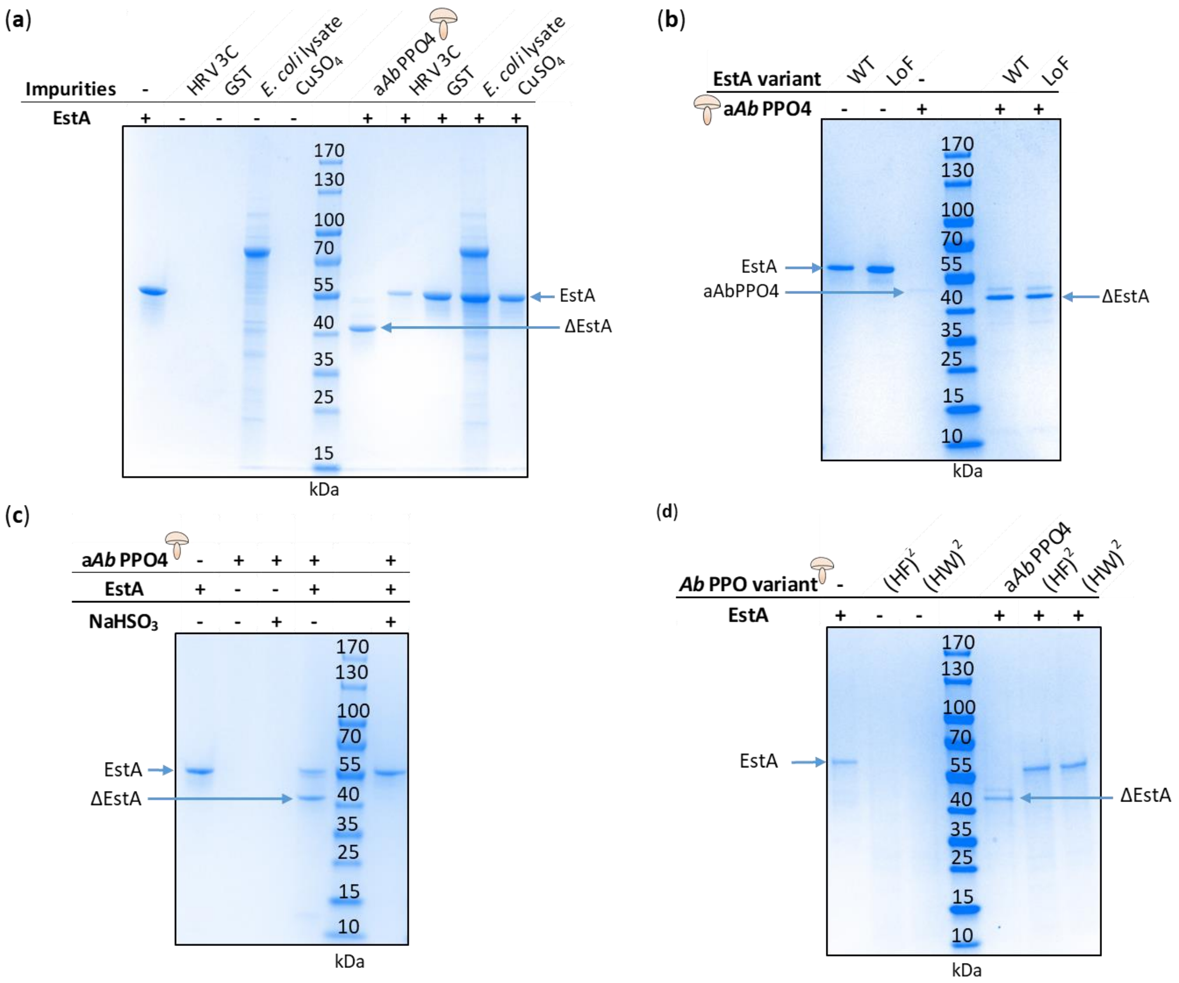

Supplementary Figure 3: **Various experiments were carried out to characterize the reaction**: Possible impurities that could influence EstA cleavage were incubated with EstA (**a**). Autoproteolysis activity of wild type (WT) EstA was excluded by cleavage of a loss-of-function (LoF) variant of EstA (**b**). Inhibition of tyrosinase activity with an irreversible inhibitor (**c**) and loss-of-function *Ab*PPO4 variants (HF)^2 and (HW)^2 (**d**) proved involvement of tyrosinase activity in the cleavage. PeqGold IV was used as a marker and sizes are given in kDa.

Activity determination of EstA_S182A

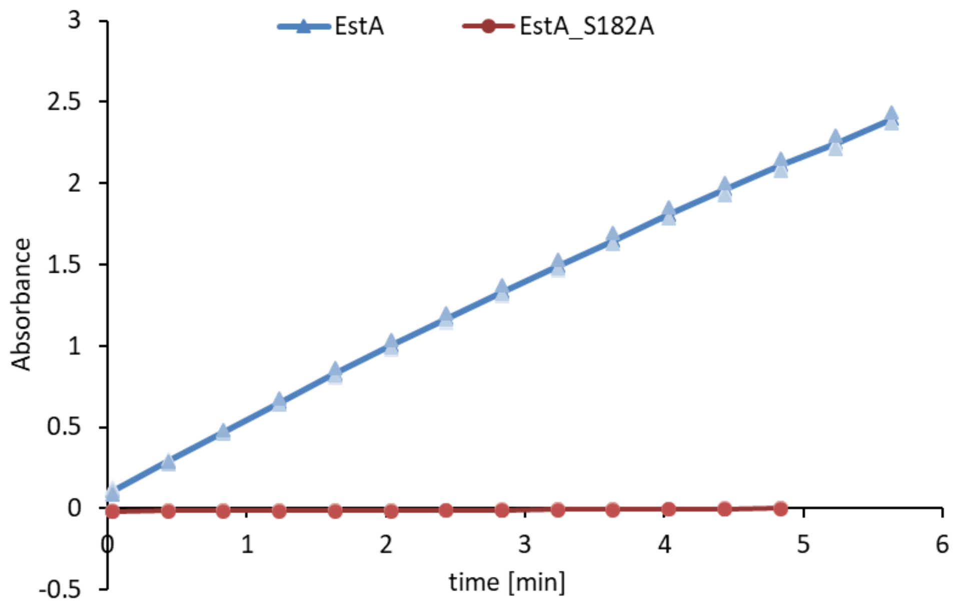

Supplementary Figure 4: **Activity of Loss-of-Function EstA variant (EstA_S182A)** compared to wild type EstA on model substrate 15.75 mM *p*NPB. Measurements were performed in triplicates. Individual data-points are shown in lighter colour, dark colour with line depicts the average.

Activity determination of a*Ab*PPO4 after inhibition

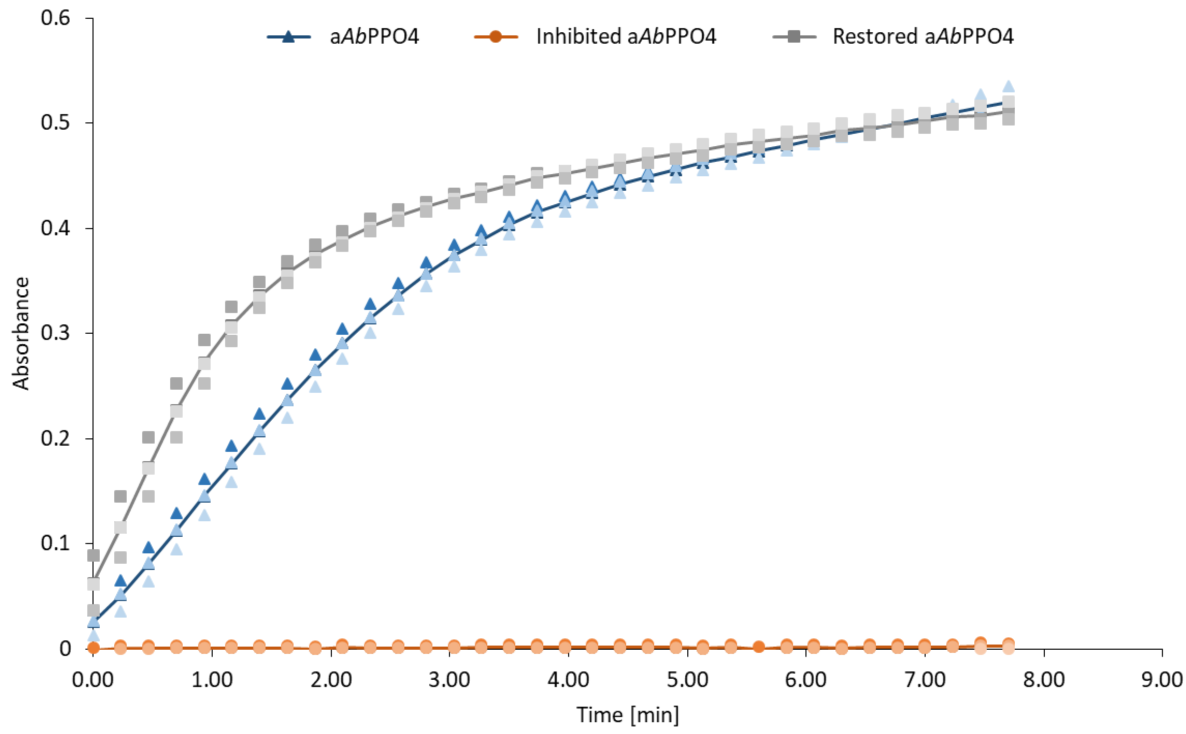

Supplementary Figure 5: **Tyrosinase activity** on 15 mM dopamine of a*Ab*PPO4 (in *blue*), inhibited a*Ab*PPO4 (in *orange*) and restored a*Ab*PPO4 (in *grey*). Restored a*Ab*PPO4 refers to a sample of inhibited a*Ab*PPO4 where fresh a*Ab*PPO4 was added to prove that sodium bisulphite was quantitatively removed, as any remains would inhibit the activity of fresh a*Ab*PPO4 as well. Measurements were performed in triplicates. Individual data-points are shown in lighter colour, dark colour with line depicts the average.

Active site of a*Ab*PPO4, a*Md*PPO2, a*Jr*TYR and a*Cg*AUS1

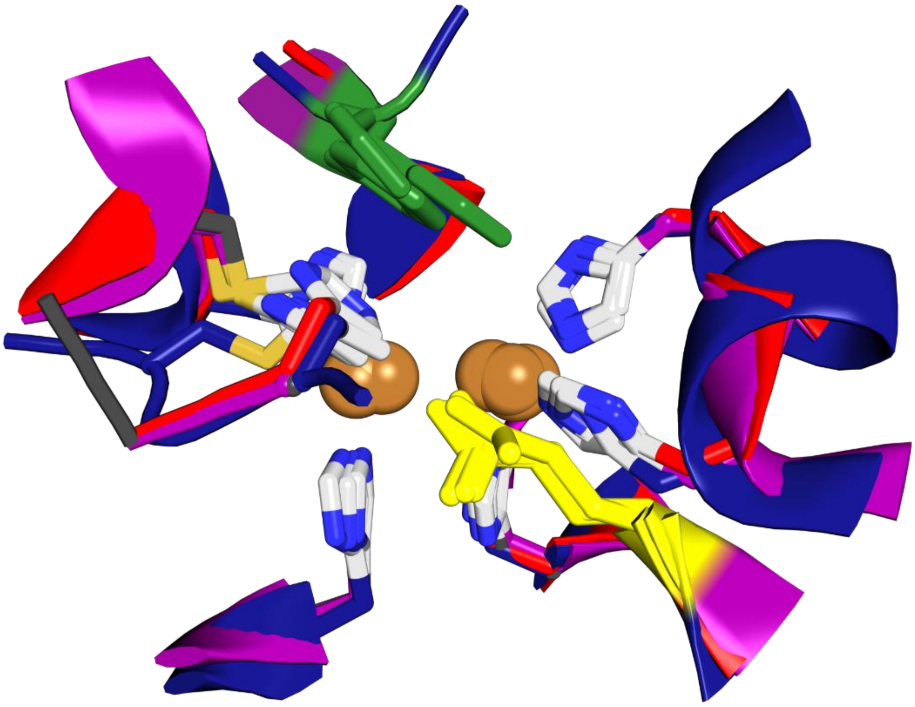

Supplementary Figure 6: **Overlay of the active centres of the four tested PPOs:** the side chains immediately surrounding the active centre are displayed as sticks and the main chains of amino acids within 8.5 Å of either copper ion as cartoon. The colours correspond to the four PPOs as follows: a*Ab*PPO4 (PDB: 5M6B): dark blue, a*Md*PPO2 (homology model): red, a*Cg*AUS1 (PDB: 4Z0Y): lilac and a*Jr*TYR (PDB 5CE9): grey. The six histidines coordinating the two copper ions are depicted in light grey (carbon) and blue (nitrogen), the yellow amino acid in front is the almost perfectly conserved glutamic acid at the active centre of nearly all PPOs ("water keeper"), the green amino acid on top of the image is the so-called gatekeeper which sits atop CuA and is a phenylalanine for plant PPOs (and an alanine for *Ab*PPO4) while at the left side the thioether-linkage of a CuA-coordinating histidine and an adjacent cysteine can be seen for all four PPOs.

Tyrosinase mutants

*Supplementary Table 4: **List of Primers for tyrosinase mutants***

Designation	***Sequence (5' - 3')***
pENTRY-lAbPPO4_fwd	agcggctcttcaatgTCTCTGCTCGCTACTGTTGG
pENTRY-lAbPPO4_rev	agcggctcttctcccTTAAGTGAGACCGATGTTGACTACAC
AbPPO4_H91F_fwd	TCCGACTTGGttCAGGGTTTATG
AbPPO4_H91F_rev	AAGAGAACTTGGCTGTGG
AbPPO4_H251F_fwd	GGAAGCTGTCttTGATGACATTCACG
AbPPO4_H251F_rev	AAACTGTTCGCATGAGTATC
AbPPO4_H57W_fwd	TTCTGGTATCtggGGTTTGCCTTTCAC
AbPPO4_H57W_rev	AGTTGGAAAAAAGAGCTGTAG
AbPPO4_H282W_fwd	CTTCTGGTTGtggCACTCCAACGTCGACC
AbPPO4_H282W_rev	ATGGGGTCAAAGGCGGCA
pGEX-6P-1_C4295A_fwd	TGTTGCCCGTaTCACTGGTGA
pGEX-6P-1_C4295A_rev	GCTGATTGCCCTTCACCG
pGEX-6P-1 -> pGEX-6P-SG_fwd	gggcgtctcagggaGTCACAGCTTGTCTGTAAGC
pGEX-6P-1 -> pGEX-6P-SG_rev	gggcgtctctcattGGCCCCTGGAACAGAACT

Extinction coefficient for dopamine

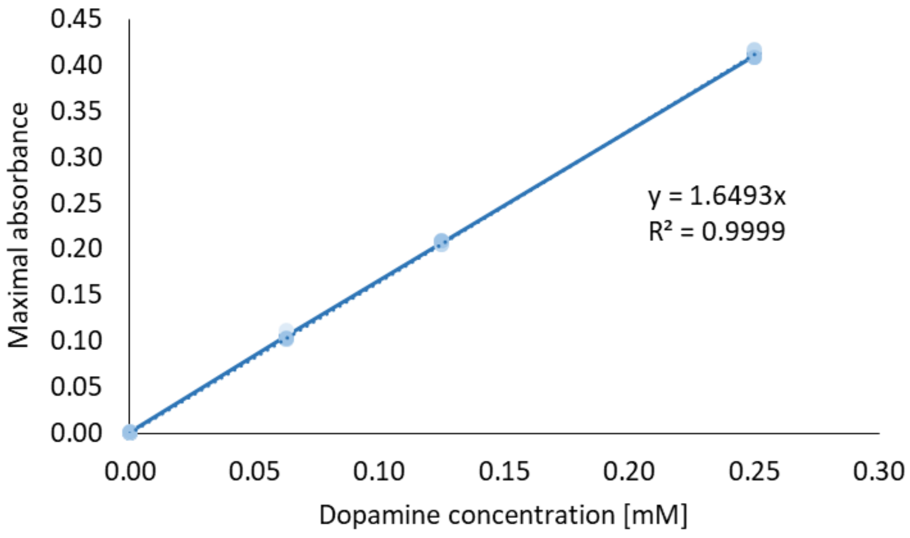

Supplementary Figure 7: **Extinction coefficient analyses of dopamine:** Maximal absorbances at 475 nm of the immediate oxidation product of dopamine at different concentrations in 0.1 M Tris-HCl pH6 buffer plotted against the concentration of dopamine. The slope of the linear portion was calculated for a least square fit trend line. The slope divided by the path length corresponds to the extinction coefficient ε_{475} = 1.6493 mM^{-1} / 0.5259 cm = 2.925 mM^{-1} cm^{-1}. Measurements were performed in triplicates. Individual data-points are shown in lighter colour, dark colour with line depicts the average.

Supplementary References

1. PeptideCutter. Available at: https://web.expasy.org/peptide_cutter/. (Accessed: 9th October 2019)
